# Carbon Restrains the Precipitation of Cu-Rich Nanoparticles in CuFeMnNi HEAs

**DOI:** 10.3390/nano15161223

**Published:** 2025-08-11

**Authors:** Mingze Wang, Mengyuan He, Yongfeng Shen, Wenying Xue, Zhijian Fan

**Affiliations:** 1 Key Laboratory for Anisotropy and Texture of Materials (Ministry of Education), School of Materials Science and Engineering, Northeastern University, Shenyang 110819, China; 2310204@stu.neu.edu.cn (M.W.); 2400652@stu.neu.edu.cn (M.H.); 2 State Key Laboratory of Digital Steel, Northeastern University, Shenyang 110819, China; xuewy@ral.neu.edu.cn; 3 Key Laboratory of Neutron Physics and Institute of Nuclear Physics and Chemistry, China Academy of Engineering Physics, Mianyang 621999, China; fanzhijian@caep.cn

**Keywords:** high-entropy alloy, Cu-rich nanoparticles, carbon, diffusion

## Abstract

In this study, we report a strategy to suppress the formation of large Cu-rich particles by adding excessive interstitial carbon into CuFeMnNi high-entropy alloys. With the increase in C contents in the CuFeMnNi HEAs annealed at 1000 °C, the size and area fraction of the submicron Cu-rich particles markedly decreased. Of note, the CuFeMnNi 1.5 at. %C alloy containing nanosized Cu-rich particles (13 nm) displayed excellent strength–ductility synergy, with yield strength of 695 ± 10 MPa, ultimate tensile strength of 925 ± 20 MPa, and ductility of 21.5%. This is because the addition of carbon significantly increases the diffusion speed of Cu atoms, thereby restraining the growth of Cu-rich nanoparticles. As a result, the comprehensive mechanical properties of the prepared HEAs were significantly enhanced. Additionally, the active diffusion channels induced by high-temperature short-time annealing significantly inhibited the grain growth, which improved the ductility. This work creates a new strategy for solving the dilemma caused by the large Cu-rich particles in the Cu-containing HEAs.

## 1. Introduction

The appearance of high-entropy alloys (HEAs) broke through the fixed thinking reflected in traditional alloy design and opened up a broader space for this discipline [1,2]. These alloys comprise at least four principal elements, with each element ranging from 5 at. % to 35 at. %, and exhibit several special intrinsic characteristics, for example, high configuration entropy, sluggish atomic diffusion, severe lattice distortion, and the “cocktail” effect [3]. These unique features endow HEAs with diversified properties, such as high strength, radiation resistance, high temperature softening resistance, etc., thereby showing great application prospects. Additionally, Cu-containing HEAs have attracted extensive attention because of their superior conductivity, thermal conductance, and crack propagation resistance. However, the addition of Cu can easily lead to Cu segregation and the precipitation of large Cu-rich particles, thereby causing the deterioration of alloy properties (especially mechanical properties) and restricting its applications [4,5].

Among the HEAs, a series of Cu-containing HEAs (CoCrFeNiCu_0.3_ [6], AlCoCrFeNiCu_0.5_ [7], AlCrFeNi_3_Cu_x_ (*x* = 0, 0.2, 0.4, 0.6, 0.8, and 1.0) [8], CuFeCrCoNi [9], CoCrCuNi [10], etc.) have been developed in recent years, receiving extensive attention. This is mainly because the more HEAs with microstructure with promising applications and outstanding comprehensive mechanical properties [11]. To address this, our study strategically reduces carbon while preserving the alloy matrix. This approach suppresses the formation of coarse Cu-rich nanoparticles without compromising precipitation strengthening effects, ultimately enhancing the alloy’s yield strength. For example, Cai et al. [12] reported that Cu segregation easily occurred in the grain boundaries of FeCoCrNiCu_x_ HEA, which caused a significant reduction in corrosion resistance due to the large potential difference between the alloy matrix and the Cu-rich phase in FeCoCrNiCu_x_ HEA. Zhu et al. [13] found that more Cu segregations formed with the increase in Cu content in as-cast AlCoCrFeNiCu_x_ HEAs, resulting in a decrease in ductility. In contrast, Gao et al. [14] reported a large number of submicron Cu-rich particles at grain boundaries and inside the interior grains of CuFeMnNi HEA, which effectively increased the strength of the HEA but greatly reduced its plasticity. To date, there has been little research that focuses on eliminating the phenomenon of Cu segregation and submicron Cu-rich particles formed in Cu-containing HEAs. This research is expected to promote the development of Cu-containing HEAs.

Noteworthily, our previous study found that large-sized Cu-rich nanoparticles are easy to form at grain boundaries and inter-dendrites in high C-content CuFeMnNi HEAs (C: 2.7 at. %) [15], which is mainly owing to the high mixing enthalpy between Cu and other constituent elements, leading to the deterioration of Cu-containing HEA’s tensile properties. Although C atoms acting as interstitial elements can introduce local lattice distortion that inhibits the nucleation of copper-rich nano-precipitates and promote the complete dissolution of Cu atoms, this condition slows grain-boundary crack propagation during deformation. Consequently, precipitation strengthening is markedly weakened, causing a significant reduction in the yield strength. Hence, this study strategically reduces C content in the CuFeMnNi matrix, thereby suppressing the formation of large Cu-rich nanoparticles without sacrificing the precipitation of the strengthening effect, thus enhancing the yield strength.

In this study, a simple and reliable method of doping interstitial C in fully recrystallized CuFeMnNi HEA is proposed to inhibit the precipitation of submicron Cu-rich particles. The strategy further reveals the influence of the introduction of C on the atomic diffusion and precipitation of the alloy, which may pave the way for developing a wide range of Cu-containing HEAs.

## 2. Experiment and Simulation

### 2.1. Materials

Equiatomic CuFeMnNi HEAs with different contents of carbon (0.15, 1.5, and 2.7 at. %C) were prepared by arc melting high purity elements (above 99.9 at. % in purity) and Fe-5 wt. %C master alloy in a vacuum induction furnace protected with a pure Ar gas atmosphere. The ingots were melted four times to ensure the homogeneity of the chemical compositions. The C fraction was measured via a Leco analyzer, and the error was within ± 0.1 at. % at nominal levels. The as-cast ingots were hot-rolled at 950 °C until the thickness was reduced by 50% (from 10 mm to 5 mm) to eliminate any defects. Subsequently, the resulting plates were homogenized at 980 °C for 2 h in Ar, followed by air-cooling. Then the homogenized sheets were cold rolled to a thickness of 1.75 mm, with a reduction of 65%. Finally, the cold-rolled sheets were annealed at 1000 °C for 5 min, followed by air-cooling. According to the carbon concentrations, the resultant materials were named 0.15C-1000-5, 1.5C-1000-5, and 2.7C-1000-5 alloys, respectively. Similarly, the CuFeMnNi HEAs annealed at 1000 °C for 240 min were named 0.15C-1000-240 and 2.7C-1000-240 alloys.

### 2.2. Characterization

Microstructure characterizations were conducted on a JEOL JSM-7001 F scanning electron microscope (SEM) equipped with an Oxford Ltd. energy dispersive X-ray spectrometer (EDS). SEM specimens were mechanically polished and subsequently electropolished in a mixture of 90% acetic acid and 10% perchloric acid at room temperature at 14 V for 15 s. Transmission electron microscopy (TEM) observations were applied at an accelerating voltage of 200 kV using a JEOL JEM-2100 F TEM combined with EDS. The alloy’s chemical compositions were determined using a JEOL JXA-8530F electron probe micro analyzer (EPMA). The specimens for TEM observations were ground to ~50 μm thickness and subsequently thinned using a Gatan 691 Precision Ion Polishing System. Multiple experiments were performed for each distinct processing condition to ensure reproducibility. Dog-bone-shaped tensile specimens with gauge dimensions of 15 × 5 × 1.5 mm^3^ were machined from the middle of the as-prepared HEAs along the rolling direction using a Borei DK7735 electron sparking machine. Uniaxial tensile tests were conducted at room temperature on a Shimadzu AG-X plus computer-controlled mechanical testing system, employing a constant strain rate of 1.0 × 10^−3^ s^−1^. Strain calibration during loading was achieved using a contactless MTS LX300 laser extensometer. Three parallel tests were performed for each sample condition.

### 2.3. Simulation

Molecular dynamics simulations of atomic diffusion in CuFeMnNi HEAs with different C content (C-free, 1.5 at. %, and 2.7 at. %C) during annealing were performed via LAMMPS [16], and the simulation results were visualized with OVITO 3.1.3 software [17]. The alloy models were created by uniformly distributing Cu, Fe, Mn, Ni, and C atoms in the model material. The overall size of the initial simulation model is 150 Å × 200 Å × 100 Å, with a total of 514,499 atoms. Periodic boundary conditions were applied to the simulation box. Due to the fact that the reports on the interatomic potential energy of C-doped CuFeMnNi HEAs is extremely scarce, the embedded atom method (EAM) is adopted to establish the interatomic potential energy of multiple atoms to describe the interaction between metal atoms [18].

**Figure 1:**
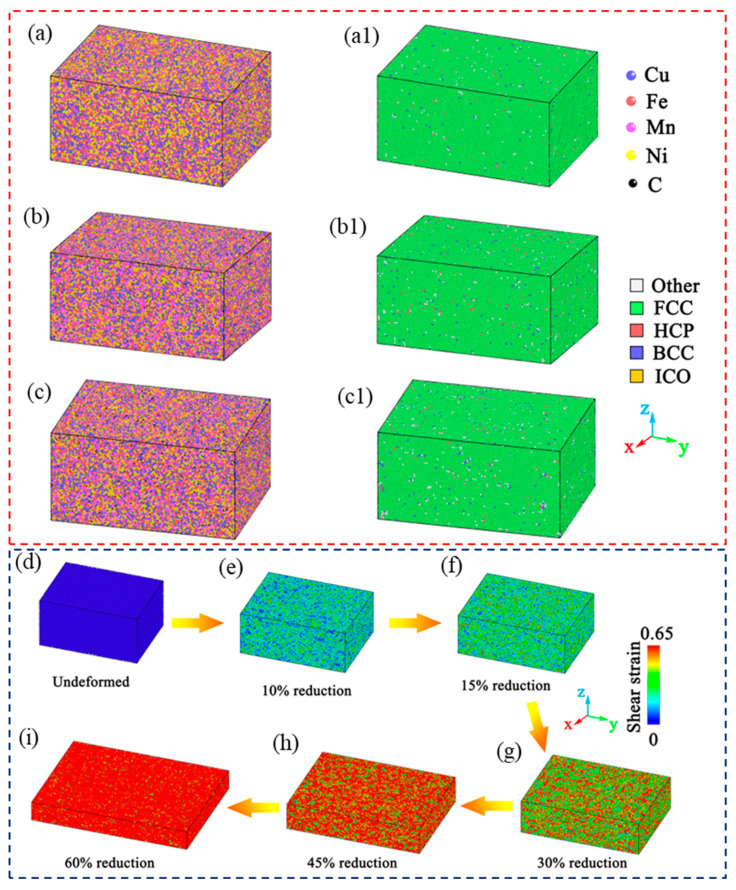
Models and crystal structure analysis of CuFeMnNi HEAs with different C concentrations after energy minimization and relaxation. (**a**,**a1**) CuFeMnNi HEA; (**b**,**b1**) CuFeMnNi-1.5 at. %C HEA; (**c**,**c1**) CuFeMnNi-2.7 at. %C HEA. (**d**–**i**) Shear strain distribution of CuFeMnNi HEAs with different C concentrations during deformation.

The interaction of short-ranged C–C atoms was described by the adaptive intermolecular reactive empirical bond order (AIREBO) potential [19]. Meanwhile, the long-range interactions between C and metal atoms were modeled using the Lennard-Jones (LJ) potential, which provides an effective approach for computing Coulombic pairwise interactions for different atoms [20]. The conjugate gradient algorithm was used to minimize the energy of the initial models of these three alloys, eliminating the overlap of atoms, and then was fully relaxed in the 300 K and isothermal isobaric system so as to obtain a balanced and stable state (Figure 1a–c). To simulate the atomic diffusion, the models first applied an external force along the z-axis, placing a large strain to roughly replace cold rolling (Figure 1d–i), and then raised the temperature from 27 °C (300 K) to 1000 °C (1273 K) over 5 ps, maintained the temperature at 1000 °C for 50 ps, and calculated the diffusion mean square displacement of each substitutional atom during the holding process. Annealing process simulation was performed in the NVT (constant number of atoms, volume, and temperature), and the time step was 0.001 ps.

## 3. Results

### 3.1. Effect of C on the Precipitation of Submicron Cu-Rich Particles

**Figure 2:**
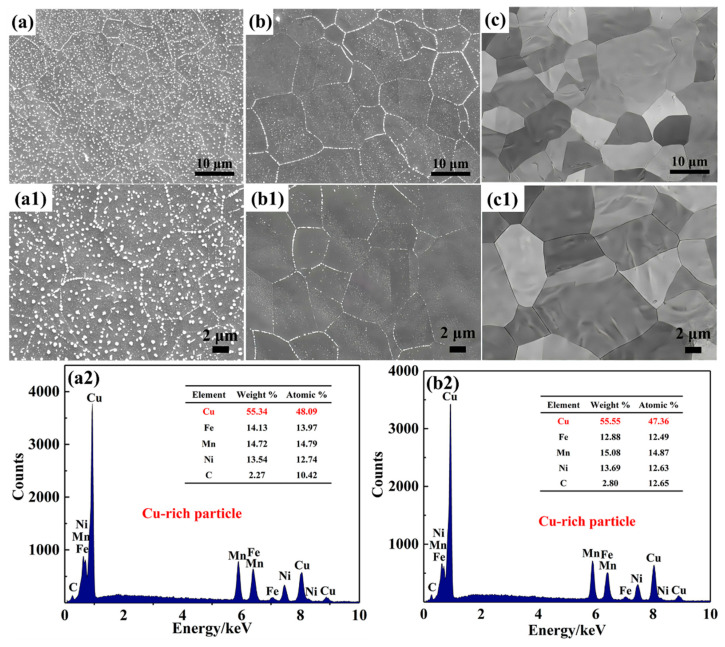
BSE micrographs of 0.15C-1000-5 (**a**,**a1**), 1.5C-1000-5 (**b**,**b1**), and 2.7C-1000-5 alloys (**c**,**c1**). EDS revealing the fine precipitates in 0.15C-1000-5 (**a2**) and 1.5C-1000-5 (**b2**) alloys.

Figure 2 shows the microstructure of 0.15C-1000-5 and 2.7C-1000-5 alloys. It can be seen that a large number of fine white precipitates occur in 0.15C-1000-5 (Figure 2a) and 1.5C-1000-5 alloys (Figure 2b), and multiple EDS energy spectrum point analysis confirmed that the white precipitates are Cu-rich particles, which mainly consist of Cu, accompanied by a few Fe, Mn, Ni, and C atoms (Figure 2(a2,b2)).

Specifically, numerous Cu-rich particles in the 0.15C-1000-5 alloy mainly distribute along grain boundaries and inside the grain interiors, and similar phenomena have been observed in the C-free CuFeMnNi HEAs [15]. When the carbon content reaches 2.7 at. %, no submicron Cu-rich particles can be seen at the grain boundaries and inside the grain interiors, which is consistent with our previous results [15]. In general, the C atom is regarded as an interstitial atom, triggering low interfacial energy and nucleation energy barriers for Cu-rich particles, indirectly suppressing the nucleation driving force of the Cu-rich particles. Evidently, with the increase in C contents in the CuFeMnNi HEAs, the Cu-rich particles significantly decrease and eventually, completely disappear (Figure 2c,(c1)).

**Figure 3:**
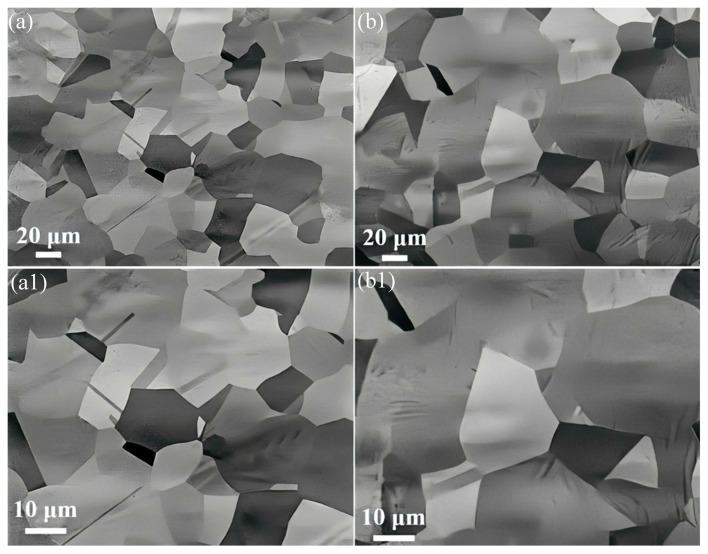
BSE micrographs of the 0.15C-1000-240 (**a**,**a1**) and 2.7C-1000-240 (**b**,**b1**) alloys.

Figure 3 shows the microstructure of the 0.15C-1000-240 and 2.7C-1000-240 alloys. Obviously, no large Cu-rich particles are precipitated at either the grain boundaries or inside the grain interiors in the two alloys, even after long-duration heat treatment. In addition, the average grain size (~36 μm) of the 2.7C-1000-240 alloy is significantly larger than that (~21 μm) of the 0.15C-1000-240 alloy, and the latter is very close to the that of the alloy annealed at the identical temperature for 5 min (Figure 2a). This indicates that the large Cu-rich particles in the 0.15C-1000-240 alloy do not rapidly dissolve into the matrix; in contrast, the dissolution process is slowly mediated by sluggish atomic diffusion during high-temperature and long-time annealing. In this process, the gradually dissolved Cu-rich particles continuously impose a strong hindering effect on the growth of grains. It is confirmed once again that the introduction of a large number of interstitial C atoms will accelerate the diffusion of Cu atoms.

**Figure 4:**
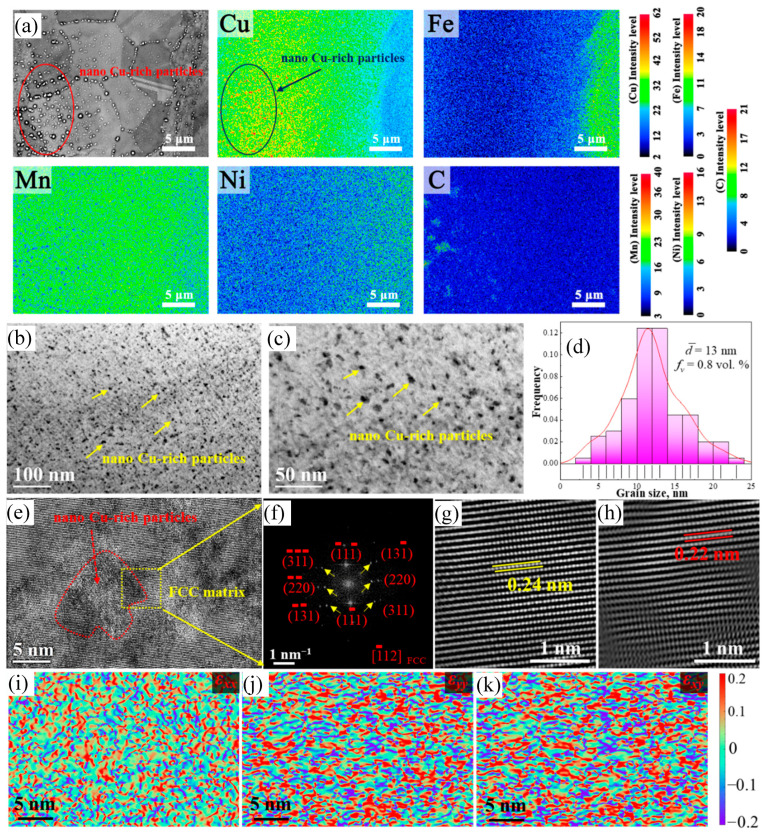
EPMA maps showing elemental distributions (**a**), TEM images (**b**,**c**), and statistical size distributions of Cu-rich nanoprecipitates in the 1.5C-1000-5 alloy (**d**), HRTEM (**e**), and the corresponding FFT in the phase interface (in yellow box) (**f**). The IFFT patterns of the matrix and Cu nanoprecipitates (**g**,**h**) and the GPA maps (**i**–**k**) show strain distributions.

EPMA was performed to reveal the compositional distribution in the 1.5C-1000-5 alloy. Element-mapping images showing the distributions of Mn, Fe, Ni, Cu, and C in the 1.5C-1000-5 alloy are provided in Figure 4a. The alloy elements are evenly distributed inside the grain interiors. Significantly, the grains contain nanoscale precipitates with Cu-rich elements. To determine the precipitates in the 1.5C-1000-5 alloy, elaborate TEM characterizations were conducted. Figure 4b,c show the TEM images of 1.5C-1000-5 alloy, distinctly showing a number of uniformly distributed Cu-rich nanoparticles in the matrix. Statistical results reveal that the mean diameter and phase volume fraction (*f_v_*) of Cu-rich nanoparticles is 13 nm and 0.8 vol. %, respectively (Figure 4d). Figure 4e,f show a HRTEM image and the corresponding FFT patterns of a Cu-rich nanoparticle neighboring matrix, respectively. The matrix taken along the <$\bar{1}$12> reveals that the 1.5C-1000-5 alloy remains the FCC phase after annealing at 1000 °C, while, second-level diffraction spots (one of them marked by a yellow arrow) can be observed from the SAED pattern taken along the <$\bar{1}$12> zonal axis for precipitate phase in the 1.5C-1000-5 alloy. The positions of these spots usually correspond to a Cu-rich nanoparticle crystal structure, which is also the FCC phase. Accordingly, the lattice parameters of the matrix and the Cu-rich nanoparticles are measured as 0.24 nm and 0.22 nm in the 1.5C-1000-5 alloy, respectively (Figure 4g,h). The related GPA deduced from Figure 4e is shown in Figure 4i–k. Here, the interface direction is selected as the *x*-axis direction, while the vertical orientation to the interface is regarded as the *y*-axis direction. The surface strain distributions from the *ε_xx_* surface show that the two phases display relatively uniform strain. Moreover, the strain of the both phases is also uniform, and no significant difference in the *ε_yy_* and *ε_xy_* surfaces. This implies that plastic deformation can occur simultaneously in the duplex-phase region, leading to uniform and continuous work hardening during plastic deformation.

### 3.2. Effect of Carbon on Diffusion of Substitutional Atoms

According to the thermodynamic theory, the atoms in the alloy continuously move. When the applied temperature is low, the atoms tend to vibrate at the equilibrium position. But when the temperature rises, the atoms may leave the original position and transition to the position with lower diffusion energy [21]. Therefore, molecular dynamics simulations were performed to calculate the mean square displacement (MSD) of each substitutional atom from its initial position for the annealing process of severely deformed CuFeMnNi HEAs with different carbon contents. This study aims to investigate how introducing interstitial C atoms influence the diffusion of the alloy’s substitutional component atoms. MSD is expressed as [22].

(1) $$\mathrm{MSD}=\langle r^{2}\left(t\right)\rangle =\frac{1}{N}\sum_{i=1}^{N}\langle {\left|r_{i}\left(t\right)-r_{i}\left(0\right)\right|}^{2}\rangle$$

Here, *r_i_*(*t*) and *r_i_*(0) are the displacement vectors of the atom at time *t* and the initial time, respectively, *N* is the total number of atoms, and the operator < > represents the ensemble average of all atoms in the whole simulation process.

According to Einstein’s diffusion law, the diffusion coefficient *D* can be expressed as [23]

(2) $$D=\underset{t\to \infty }{\lim}\frac{1}{2tN^{\prime }}\langle {\left|r\left(t\right)-r\left(0\right)\right|}^{2}\rangle$$

Here, *N*’ is the dimension of the simulation system (*N*’ = 3 for the block).

Combining Equations (1) and (2), the relationship between diffusion coefficient *D* and mean square displacement MSD can be described as

(3) $$D=\frac{\mathrm{MSD}}{6t}$$

Here, *D* can be obtained by 1/6 times the slope of the MSD-*t* curve.

**Figure 5:**
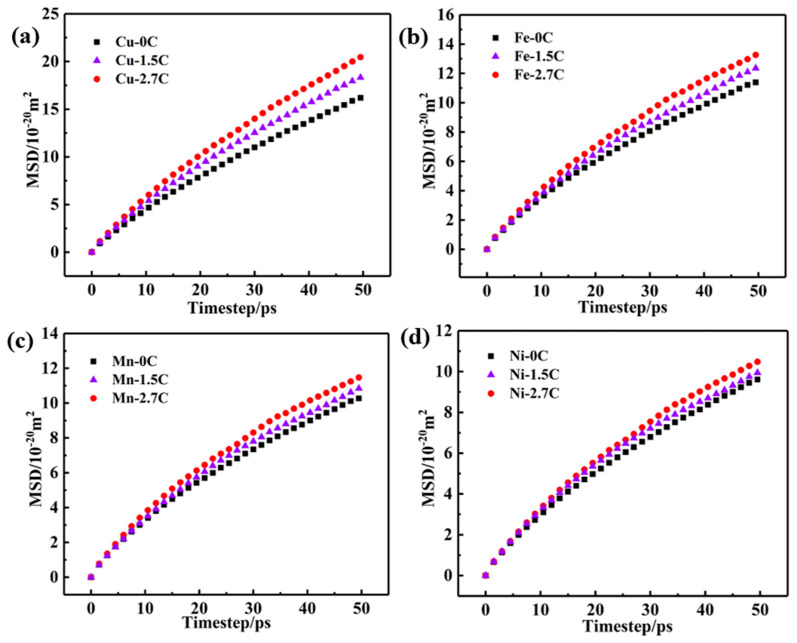
The evolution of MSD for the substitutional component atoms in the 1.5C-1000-240 and 2.7C-1000-240 alloys under varying annealing times. (**a**) Cu, (**b**) Fe, (**c**) Mn, and (**d**) Ni.

Figure 5 exhibits the evolution of the MSD values for substitutional component atoms (Cu, Fe, Mn, and Ni) in different alloys with increasing annealing durations at 1000 °C. Apparently, the MSD values of each substitutional element in the three alloys increase with the extension of holding time. This indicates that with the increase in holding time, the probability of atoms jumping is significantly enhanced, and the diffusion distance of atoms is increased. In addition, with the increase in C contents in the alloy, the MSD value of each substitutional component atom also increases. This means that the addition of C induces the increasing slope of the curves for these elements. Evidently, the addition of C increases the diffusion coefficient (*D*) of the substitutional atoms of the HEAs, thus promoting the diffusion of the substitutional atoms during high temperature annealing, which is consistent with the experimental results in Section 3.1. Moreover, the diffusion coefficient *D* of each substitutional element in the three alloys can be obtained by calculating the slopes of the respective curves, and the calculation results are shown in Table 1. It is significant that the diffusion coefficient of the Cu atoms increases from 5.12 to 6.61 with the increase in C contents, revealing that the interstitial C atoms exert a prominent promoting effect on the diffusion of Cu atoms. In particular, it is obvious that the diffusion coefficient of Cu is the largest, which means that the diffusion speed of Cu is the fastest. Furthermore, the addition of C obviously increases the diffusion coefficient *D* of the substitutional elements in the alloys, indicating that the C addition promotes the diffusion of the substitutional elements in the alloys, which is consistent with the results reported by Lukianova et al. [24]. Among these substitutional atoms, the diffusion coefficient of the Cu atoms shows a significant increment, and its diffusion speed is the fastest. Compared with the reported values [21,24,25], such a high diffusion coefficient of the substitutional elements in this study is mainly related to the fact that (i) the target model alloy in this study is set as a single crystal structure; (ii) a large number of defects are stored after severe deformation pretreatment; and (iii) the diffusion activation energy of atoms in the alloy is greatly reduced at a high temperature of 1000 °C. As a result, the substitutional atoms are very active and easily migrate and diffuse, leading to the increasing diffusion coefficient of the substitutional atoms.

**Figure 6:**
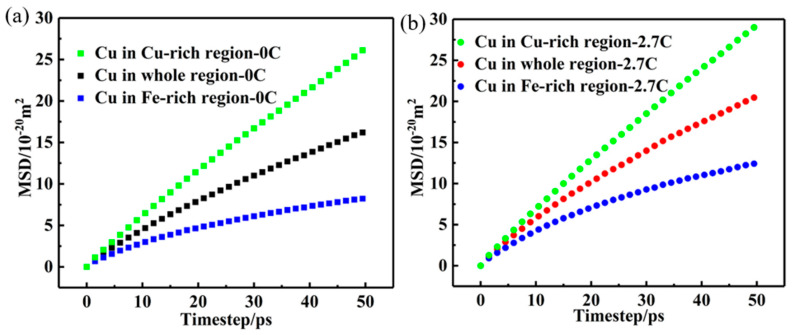
The evolution of Cu-MSD in the Cu-rich and Fe-rich regions with the holding time for the C-free (**a**) and 2.7 at. %C-doped (**b**) alloys annealed at 1000 °C.

Specifically, the target alloy displays a double FCC structure [14]; hence, the diffusion speed of Cu atoms in the Fe-rich FCC_1_ and Cu-rich FCC_2_ phases must be very different. According to the EDS results of two phases in the target alloys, the four Fe-rich and Cu-rich regions, with C (2.7 at. %) and without C, were constructed using LAMMPS, and the chemical compositions of the constructed regions are shown in Table 2. To verify the influence of Cu atomic diffusion on different phase regions in C-free and 2.7 at. %C doped CuFeMnNi HEAs, Figure 6 shows the evolution of the Cu-MSD values in Fe-rich, Cu-rich, and the entire regions with the varying holding durations at 1000 °C. It can be seen that the Cu-MSD values in the Cu-rich region rapidly increase with the increase in holding time in both undoped C and C-doped alloys, and the corresponding curves exhibit large inclination angles. In contrast, the Cu-MSD values in the Fe-rich region slowly increase with the increase in holding time, and the corresponding curve is flat. Compared with the C-free alloys, the introduction of C significantly increases the Cu-MSD values in the Cu-rich regions and Fe-rich regions, and the corresponding curves are more inclined, which means that the C addition promotes the Cu diffusion in the Cu-rich and Fe-rich regions. Subsequently, the same severe deformation as that mentioned above was applied, and the diffusion coefficients of the Cu atoms in Fe-rich and Cu-rich regions were calculated during the annealing process. The corresponding diffusion coefficients are shown in Table 3. It can be seen that the diffusion coefficients of Cu atoms increase from 8.57 to 9.57 with the increase in C concentrations, revealing that interstitial C atoms have a more prominent influence on the diffusion of Cu atoms. In summary, high-temperature annealing also increases the solid solubility of substitutional elements in the matrix so that the Cu-rich particles can be quickly dissolved into the matrix to realize homogenization.

## 4. Discussion

### 4.1. Diffusion Mechanism of Different C Concentrations

The CuFeMnNi HEAs with different C concentrations included in this study exhibit stable dual FCC structures, that is, Fe-rich FCC_1_ and Cu-rich FCC_2_ phases. Moreover, the two phases are cross-distributed, which has been proved in a previous study [15]. After cold rolling, the Cu-rich and Fe-rich phases in the alloys have severely segregated, and the Cu-rich region is surrounded by the Fe-rich region. Therefore, during the annealing process, the Cu segregation region not only needs to reduce the segregation by the diffusion of Cu-rich nanoparticles, but also requires the diffusion of Cu-rich nanoparticles to the adjacent Fe-rich region and beyond, thus increasing the diffusion difficulty. Noteworthily, when annealed at 1000 °C, the Cu atoms in the Cu segregation zone are extraordinarily active, and the abundant Cu atoms aggregate to form local clusters and cause lattice distortions, leading to the complexity of diffusion pathways and hindering atomic migration. Hence, the diffusion rate is slightly slower in the CuFeMnNi HEAs (Figure 6). As a result, the concentrations of all components in the Cu segregation region tend to be consistently quick, but the Cu concentration still exceeds the solid solubility of this region, thus diffusing to the adjacent Fe-rich region. However, due to the different radii of each substitutional element atom in the alloy, Cu (0.128 nm), Fe (0.124 nm), Mn (0.137 nm), Ni (0.125 nm), and the mixing enthalpy of Cu and other elements is large; in fact, Cu and Fe are almost incompatible. Thus, when Cu-rich nanoparticles diffuse, the vacancies formed by Fe and Ni are small, a condition unsuitable for the diffusion of Cu atoms [26]. Moreover, Cu-rich nanoparticles face strong interatomic repulsion in the Fe-rich region, making diffusion into the vacancies in the lattice difficult. The diffusion coefficient of Cu-rich nanoparticles in the Fe-rich region is 3.32 (Table 3), which is distinctly lower than the value of 9.57 in the Cu-rich region and the value of 6.61 in the whole region, leading to the slightly slow diffusion of Cu-rich nanoparticles in the Fe-rich region of the 2.7C-1000-5 alloy. Therefore, in the cold-rolled CuFeMnNi-0.15 at. %C HEA after annealing at 1000 °C for 5 min, the precipitated submicron Cu-rich nanoparticles are distributed within grains and along the grain boundaries. In the long-term heat treatment process at 1000 °C for 240 min, the diffusion rate of Cu atoms, as well as the vacancy, are greatly increased, making Cu atoms fully diffuse, promoting the dissolution of submicron Cu-rich nanoparticles, and thus eliminating the precipitation of Cu-rich nanoparticles.

**Figure 7:**
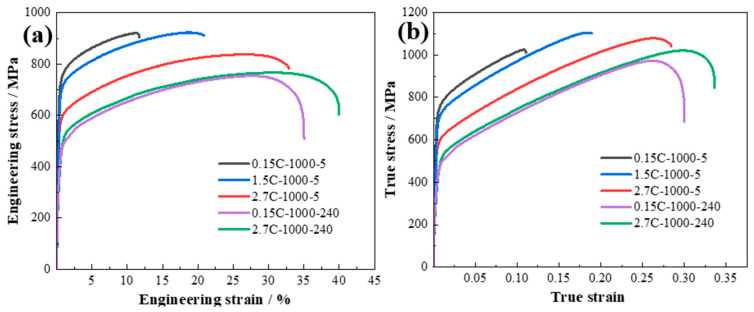
(**a**) Tensile engineering stress vs. strain (**a**) and true stress vs. strain (**b**) of the 0.15C-1000-5, 1.5C-1000-5, 2.7C-1000-5, 0.15C-1000-240, and 2.7C-1000-240 alloys, respectively.

For high-content C-doped CuFeMnNi HEA, the addition of C can reduce the energy barrier of vacancy-mediated diffusion in the alloy during high temperature annealing at 1000 °C [24,25,27], thus significantly increasing the diffusion rate of each substitutional element atom in the alloy, especially the diffusion speed of Cu atoms. In addition, it has been determined that the high C content of austenite is responsible for the transformation from γ to ε-martensite under deformation [25]. Therefore, the Cu atoms in the Cu-rich region of the alloy are more active, and the diffusion rate is faster (Figure 7). Since the Cu concentration in this region obviously exceeds the solid solubility, the Cu atoms have more driving force to diffuse to the Fe-rich region. However, due to the different affinity of Cu and Fe for C, more C atoms enter the interstitial positions in the Fe-rich region, resulting in severe lattice distortions in the Fe-rich region, which leads to the changes in vacancy concentration and size [25,28]. Thus, the active Cu atoms exhibit more opportunities to diffuse in the Fe-rich region, and the diffusion speed of Cu atoms in the Fe-rich region is improved (Figure 7). Therefore, during the high temperature annealing at 1000 °C, the addition of excessive C greatly increases the diffusion speed of Cu in the Cu-rich and Fe-rich regions of the alloy, and the diffusion distance of Cu atoms increases accordingly. Consequently, Cu atoms have more opportunities to fully diffuse to the Fe-rich region. However, owing to the incompatibility between Cu and Fe, Cu atoms are likely to continuously diffuse to the adjacent Cu-rich region, thus achieving overall homogenization. This causes the submicron Cu-rich particles to disappear completely in CuFeMnNi-2.7 at. %C HEA annealed at 1000 °C for 5 min. In particular, after long-time heat treatment at 1000 °C for 4 h, the grains are not affected by Cu-rich particles, and the grain size increases rapidly by about 26 μm. In addition, the stacking-fault energy (SFE) values of the 0.15C-1000-5, 1.5C-1000-5, and 2.7C-1000-5 alloys are calculated to be 393.8 mJ·m^−2^, 394.2 mJ·m^−2^, and 394.7 mJ·m^−2^, respectively, using JMatPro 7.0 software. Interestingly, when the C concentration increases, the SFE of the CuFeMnNi HEA slightly increases. Thus, the increasing SFE induced by long-term aging at high temperatures further inhibits precipitation behavior [29], i.e., the elevated C concentration markedly reduces the formation of precipitates owing to the increasing SFE.

### 4.2. Strengthening Mechanisms

Figure 7a displays engineering stress–strain curves of the 0.15C-1000-5, 1.5C-1000-5, and 2.7C-1000-5 alloys. Impressively, 0.15C-1000-5 alloy exhibits the highest yield strength of 700 MPa and ultimate tensile strength of 917 MPa, while retaining a good ductility of 11%. When the C content increases, the ductility of CuFeMnNi HEAs increases at the expense of strength. When the C content increases to 1.5 at. %C, 1.5C-1000-5 alloy displays excellent strength–ductility balance, with a yield strength of 695 MPa, ultimate tensile strength of 925 MPa, and ductility of 21.5%. When the C content increases to 2.7 at. %C, 2.7C-1000-5 alloy exhibits a high ductility of 33% but low yield strength of 565 MPa and ultimate tensile strength of 852 MPa, respectively. It has been noticed that there is no precipitation of Cu-rich particles in the two alloys (Figure 3). The yield strength of 0.15C-1000-240 and 2.7C-1000-240 alloys is 352 ± 10 MPa and 312 ± 10 MPa, respectively. Meanwhile, the ultimate tensile strength of 0.15C-1000-240 and 2.7C-1000-240 alloys is 718 ± 20 MPa and 715 ± 20 MPa, retaining a ductility of 35% and 41% for the 0.15C-1000-240 and 2.7C-1000-240 alloys, respectively. Obviously, when the CuFeMnNi with different C concentrations alleviated and dissipated the precipitation behavior of Cu-rich particles after long-term annealing at high temperatures, the tensile strengths of the alloys were significantly reduced. Figure 7b displays the true stress–strain curves of all the alloys, and the true stress of 1.5C-1000-5 alloy is the highest (1105 MPa) among all the alloys, which means that the former indeed exhibits superior yield and ultimate tensile strength. This is attributed to the fact that the small-sized Cu-rich nanoparticles (13 nm) can significantly enhance the work hardening ability of the 1.5C-1000-5 alloy, leading to the increase in comprehensive mechanical properties. On the other hand, the high diffusion channels induced by high-temperature short-time (5 min) annealing significantly inhibit the grain growth, thereby enhancing the ductility of the 1.5C-1000-5 alloy. This leads to the outstanding comprehensive mechanical property of the 1.5C-1000-5 alloy.

Generally, the strength of metallic materials mainly derives from the following five processes, i.e., lattice friction stress ($\sigma_{0}$), solid solution hardening ($∆\sigma_{S}$), grain boundary hardening ($∆\sigma_{G}$), dislocation hardening ($∆\sigma_{D}$), and precipitation hardening ($∆\sigma_{P}$). Here we provide a detailed introduction to the origin of strength contributions for C-doped CuFeMnNi HEAs. Basically, the yield strength is estimated by [30]

(4) $$\sigma_{y}=\sigma_{0}+∆\sigma_{S}+∆\sigma_{G}+∆\sigma_{D}+∆\sigma_{P}$$

Here, $\sigma_{0}$ = 109 MPa is the lattice friction strength of the CuFeMnNi HEA [30]. In the following text, the contributions of the other four increments to the yield strength will be discussed in detail in sequence.

For the CuFeMnNi HEA, the $\sigma_{0}$ is generally regarded as 109 MPa [30]. Furthermore, for the CuFeMnNiC_*x*_ alloy system, carbon can reside as a solute in the CuFeMnNi matrix. Thus, the contribution of C addition to $∆\sigma_{S}$ can be evaluated using a substitutional solid solution strengthening model based on dislocation–solute elastic interaction and be estimated by [31]

(5) $$∆\sigma_{S}=\frac{\mathrm{MG}\varepsilon_{S}^{3/2}c^{1/2}}{700}$$

where the key parameters include the Taylor factor M with a value of 3.06 for the FCC phase and the system shear modulus G = *E*/2 (1 + *μ*) = 77 GPa, respectively [32]. Also, the E and *μ* denote Young’s modulus and Poisson’s ratio. *C* represents the carbon percentage in the CuFeMnNi C_*x*_ HEAs. The solute–solvent interaction parameter *ε_s_* is demonstrated as

(6) $$\varepsilon_{s}=|\frac{\varepsilon_{G}}{1+0.5\varepsilon_{G}}-3\cdot \varepsilon_{a}|$$

which incorporates both elastic mismatch *ε_G_* and atomic size mismatch *ε_a_*, defined as

(7) $$\varepsilon_{G}=\frac{1}{G}\frac{\partial G}{\partial c}$$

(8) $$\varepsilon_{a}=\frac{1}{a}\frac{\partial \alpha }{\partial c}$$

where *a* is the lattice constant of FCC phase (~3.659 Å). The parameter $\varepsilon_{G}$ is usually negligible compared to $\varepsilon_{a}$. Consequently, the $∆\sigma_{S}$ of the 0.15C-1000-5, 1.5C-1000-5, 2.7C-1000-5, 0.15C-1000-240, and 2.7C-1000-240 alloys is calculated as 46 MPa 13 MPa, 15 MPa, 46 MPa, and 15 MPa, respectively.

$∆\sigma_{G}$ represents the strengthening from grain boundaries and is computed via the classical Hall–Petch relationship [31]:

(9) $${∆\sigma }_{G}=\;kd^{-1/2}$$

Here, *k* is the Hall–Petch coefficient (494 MPa μm^1/2^ for the CuFeMnNiCo HEAs [15]); *d* is the average grain size. Thus, ∆*σ*_*G*_ can be expressed in terms of the “rule of mixture”. The average grain sizes of the 0.15C-1000-5, 1.5C-1000-5, 2.7C-1000-5, 0.15C-1000-240, and 2.7C-1000-240 alloys are 10.1, 7.3, 12.5, 21.6, and 40.5 μm, respectively. Consequently, the calculated $∆\sigma_{G}$ is 156, 187, 143, 110, and 78 MPa.

After annealing at 1000 °C for 5 min, abundant dislocations are observed in the 0.15C-1000-5 and 2.7C-1000-5 alloys, causing the interactions and entanglements of dislocations. As a result, the glide dislocations are effectively trapped during plastic deformation, leading to an increase in the yield strength. The strength contributed by the accumulated dislocations can be calculated via the Taylor hardening model [31]:

(10) $${∆\sigma }_{D}=\;M\alpha \mathrm{Gb}\rho^{1/2}$$

Here, the associated parameters include Taylor factor *M* = 3.06, empirical FCC constant *α* = 0.2, shear modulus *G* = 77*G*, Burgers vector *b* = 0.258, and dislocation density *ρ,* determined by the following Equation [32]:

(11) $$\;\rho \;=2\sqrt{3}\cdot \varepsilon /\left(D_{v}b\right)$$

Here, *ε* and *D_v_* are the micro strain and the average crystallite domain size, respectively. The micro strain of each alloy can be acquired from the (111), (200), and (220) diffraction peaks based on the assumption of the Cauchy-type function [31]:

(12) $$\beta \cos\theta =K\lambda /D_{v}+\left(4\sin\theta \right)\cdot \varepsilon$$

in which the related parameters include XRD peak broadening *β*, Bragg angle *θ* for individual peaks, dimensionless constant K = 0.9, and Cu K_α_ radiation wavelength *λ* = 0.15405 nm. Using Origin 2019 software, Gaussian functions were fitted to the (111), (200), and (220) diffraction peaks of the FCC matrixes for the alloys annealed at various temperatures to extract the β and θ values. Subsequently, the *β*cos*θ* vs. 4sin*θ* plots were drawn based on Williamson–Hall analysis, in which crystallite size *D_v_* and strain *ε* are determined from the intercept and slope, respectively. Hence, *D_v_* of the 0.15C-1000-5, 1.5C-1000-5, 2.7C-1000-5, 0.15C-1000-240, and 2.7C-1000-240 alloys is 13 nm, 363 nm, 18 nm, 341 nm, and 348 nm, respectively. Accordingly, ∆*σ_D_* is evaluated as 307 MPa, 115 MPa, 307 MPa, 115 MPa, and 116 MPa.

$∆\sigma_{p}$ in the CuFeMnNi alloys with different C concentrations mainly originated from Cu-rich nanoprecipitates. The underlying mechanism is Orowan strengthening [15]. The dominant strengthening approach is the Orowan bypass mechanism, which can be predicted via the Ashby–Orowan Equation [32]:

(13) $$∆\sigma_{\mathrm{Orowan}}=\left(0.538\mathrm{Gb}f_{l}^{\;1/2}/D\right)\ln\left(D/2b\right)$$

where *f_l_* is the volume fraction, and *D* is the average diameter of precipitates, respectively. On the basis of the previous analysis, there is no precipitation phase in the 2.7C-1000-5, 0.15C-1000-240, and 2.7C-1000-240 alloys. Thus, the precipitation strengthening can be ignored in the three alloys, and the $∆\sigma_{p}$ is 124 and 298 MPa for the 0.15C-1000-5 and 1.5C-1000-5 alloys, respectively.

**Figure 8:**
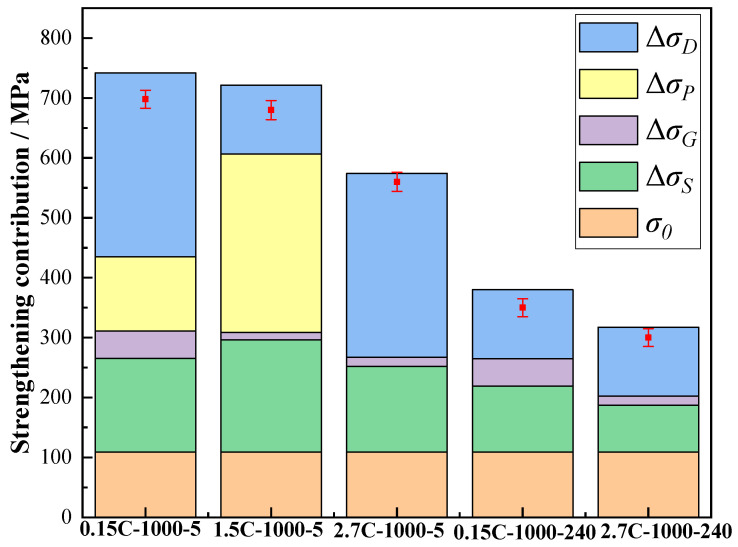
The contributions of various strengthening mechanisms to the yield stresses of the five alloys; for comparison, the measured values are shown as red dots.

The strength contributions from the five items are shown in Figure 8. For the 2.7C-1000-5, 0.15C-1000-240, and 2.7C-1000-240 alloys, the strength contributions to the yield strength are mainly derived from dislocation strengthening. This is attributed to the large grain size generating numerous dislocations slip paths. Specifically, for the 0.15C-1000-5 and 1.5C-1000-5 alloys, the key strength contributions are precipitation strengthening and dislocation strengthening. The 0.15C-1000-5 alloy can introduce precipitation strengthening via the precipitation of the Cu-rich particles. However, the large Cu-rich particles, with an average size of 430 nm, require higher dislocation stress to bypass them. This results in an increase in the yield strength by sacrificing ductility. Nevertheless, the 1.5C-1000-5 alloy, with fine precipitates (13 nm), shows extraordinary precipitation strengthening in comparison with dislocation strengthening. In general, the introduction of appropriate interstitial C atoms effectively enhances the diffusion capability of the 1.5C-1000-5 alloy, which can not only induce the presence of small-sized Cu-rich nanoparticles, but also significantly reduce the grain size of the matrix. That is to say, the Cu-rich nanoparticles significantly improve the work hardening ability of the alloys, ensuring their strength. In addition, fine grain-size still serves as an effective barrier for slip transmission, significantly preserving the ductility of the alloys. Similarly, the comparable scenarios and underlying mechanisms have been revealed in low-density steel [33] and low-carbon steel [34], in which the synergistic precipitation and the refinement of grains have played key roles in achieving the eminent combination of strength and ductility. Hence, the precipitation of Cu-rich nanoparticles (13 nm) can not only reduce the brittleness of 1.5C-1000-5 alloy, but also enhance work hardening ability during deformation. This can enhance the comprehensive mechanical properties of the 1.5C-1000-5 alloy. Simultaneously, as shown in Figure 6, the diffusion coefficient of Cu atoms in the CuFeMnNi matrix gradually elevates from 5.12 to 6.61 when the C concentrations increase. This is attributed to the gradual reduction in activation energy inside the CuFeMnNi HEA matrix, which effectively decreases the energy barrier for the thermal motion of Cu atoms while promoting the solid solution. Consequently, the higher C content (2.7 at. %) accelerates the solid solution of Cu atoms in the 2.7C-1000-5 alloy, thereby significantly suppressing the precipitation of Cu-rich particles in the 2.7C-1000-5 alloy. Accordingly, precipitation strengthening is significantly reduced, leading to a drop in the yield strength. Recent studies on the HEAs have demonstrated that dispersedly distributed precipitates significantly enhance the yield strength by contributing to both precipitation strengthening and dislocation strengthening [29,30]. Nevertheless, we demonstrate that a preferential effect can be achieved at an optimal C content of 1.5 at. %, in which the formation of fine Cu-rich nano-precipitates (13 nm) is promoted, and excessive growth of larger Cu-rich nano-precipitates is effectively inhibited. This mechanism can jointly enhance both precipitation strengthening and grain refinement, significantly improving the yield strength of the 1.5C-1000-5 alloy.

In summary, the 1.5C-1000-5 alloy with nanosized Cu-rich precipitates exhibits exceptionally broad application prospects, showing eminent potential in fields requiring high electrical/thermal conductivity and corrosion-resistant service environments, as well as for applications in new energy technologies.

## 5. Conclusions

In this study, the effect of C alloying on the precipitation of submicron Cu-rich particles in the CuFeMnNi HEAs is explored in detail through experiments and molecular dynamics simulation. The main results are summarized as follows:

(1) With the increase in C content in the CuFeMnNi HEAs, the size and area fraction of the precipitated Cu-rich nanoparticles decrease significantly, finally disappearing completely.
(2) After holding the alloy at 1000 °C for 4 h, no large Cu-rich particles were found in the C-doped CuFeMnNi HEAs, while the grain growth of the 0.15-1000-5 alloy is extremely sluggish (increased by ~4 μm) because of the slow dissolution of Cu-rich nanoparticles and their continuous pinning effect on grain boundaries.
(3) During the high-temperature annealing at 1000 °C, the excessive C greatly increases the diffusion speed and diffusion distance of Cu atoms in the C-doped CuFeMnNi HEAs. Thus, Cu atoms have more opportunities to fully diffuse to the Fe-rich region (and even to the adjacent Cu-rich region) to achieve homogenization, thereby inhibiting the precipitation of Cu-rich nanoparticles.
(4) The increasing carbon content enables an extraordinary ductility of 33%. The 1.5C-1000-5 alloy displays a superior strength–ductility combination, with the yield strength of 695 ± 10 MPa, ultimate tensile strength of 925 ± 20 MPa, and ductility of 21.5%. These results are attributed to the fact that the Cu-rich nanoparticles can significantly elevate the work hardening ability of the 1.5C-1000-5 alloy. In addition, the high diffusion channels induced by short-time annealing significantly inhibit the grain growth, leading to an increase in the ductility.

## Figures and Tables

**Table 1:** Diffusion coefficients of substitutional component atoms in the CuFeMnNi, 1.5C-1000-240, and 2.7C-1000-240 alloys.

Alloy	Diffusion Coefficient/10^−10^m^2^·s^−1^			
	Cu	Fe	Mn	Ni
CuFeMnNi	5.12	3.62	3.23	3.04
1.5C-1000-240	5.93	3.93	3.44	3.13
2.7C-1000-240	6.61	4.24	3.64	3.35

**Table 2:** Chemical compositions of constructed Fe- and Cu-rich regions, with and without C (at. %).

C Content/at. %	Region	Chemical Compositions/at. %				
		Cu	Fe	Mn	Ni	C
0C	Fe-rich region	12	41	25	22	-
	Cu-rich region	41	12	25	22	-
2.7C	Fe-rich region	11.3	40.5	24	21.5	2.7
	Cu-rich region	40.5	11.3	24	21.5	2.7

**Table 3:** Diffusion coefficients of Cu in the C-free and C-doped Cu-rich, Fe-rich, and the entire region at 1000 °C.

C Content/at. %	Diffusion Coefficient/10^−10^m^2^·s^−1^		
	Cu-Rich Region	Entire Region	Fe-Rich Region
0	8.57	5.12	2.13
2.7	9.57	6.61	3.32

## Data Availability

All data used in this study are available upon request from the corresponding author.
